# Ameliorative effects of Xue-Fu-Zhu-Yu decoction, Tian-Ma-Gou-Teng-Yin and Wen-Dan decoction on myocardial fibrosis in a hypertensive rat mode

**DOI:** 10.1186/s12906-016-1030-3

**Published:** 2016-02-06

**Authors:** Guohua Zhang, Guang Yang, Yan Deng, Xiangling Zhao, Yingbao Yang, Jinjun Rao, Wenya Wang, Xin Liu, Jian He, Lin Lv

**Affiliations:** 1 School of Traditional Chinese Medicine, Southern Medical University, 1838 Guangzhou Avenue North, Guangzhou, P. R. China; 2 School of Pharmaceutical Sciences, Southern Medical University, 1838 Guangzhou Avenue North, Guangzhou, P. R. China

**Keywords:** Xue-Fu-Zhu-Yu decoction, Tian-Ma-Gou-Teng-Yin, Wen-Dan decoction, Hypertension, Myocardial fibrosis, SHR, TGF-β_1_

## Abstract

**Background:**

Xue-Fu-Zhu-Yu decoction (XFZYD), Tian-Ma-Gou-Teng-Yin (TMGTY) and Wen-Dan decoction (WDD) are Chinese herbal formulas used to treat hypertension and cardiovascular diseases in traditional Chinese medicine (TCM). The goal of our study is to determine if XFZYD, TMGTY or WDD treatment ameliorated myocardial fibrosis in spontaneously hypertensive rats (SHRs) and to identify the mechanisms underlying any beneficial effects observed during the courses of the investigation.

**Methods:**

Forty-five 12-week-old male spontaneously hypertensive rats and five age-matched male Wistar-Kyoto control rats were studied for 16 weeks. Each day 6 g∙kg^−1^ or 12 g∙kg^−1^ of XFZYD, TMGTY or WDD was orally administered at the indicated dose, and the systolic blood pressure (SBP) of all rats was measured using the tail-cuff method. Collagen levels were measured via hydroxyproline content assays and histological examination. Transforming growth factor beta-1 (TGF-β_1_) protein levels were determined via immunhistochemical and Western blot analysis. TGF-β_1_ mRNA levels were assessed using real-time reverse transcription polymerase chain reaction.

**Results:**

Systolic blood pressure was unaffected, but collagen and TGF-β_1_ levels in SHRs treated with captopril and XFZYD (12 g∙kg^−1^) were significantly reduced when compared with untreated control SHRs. Administration of 12 g∙kg^−1^ XFZYD increased myocardial cell protection and decreased TGF-β_1_ mRNA and protein expression when compared with the other SHR treatment groups.

**Conclusions:**

XFZYD treatment demonstrated a superior ability to reverse myocardial fibrosis when compared with WDD or TMGTY treatment in SHRs. XFZYD also decreased TGF-β_1_ mRNA and protein expression, suggesting that the TGF-β_1_ signaling pathway plays a role in the therapeutic effects of XFZYD treatment.

**Electronic supplementary material:**

The online version of this article (doi:10.1186/s12906-016-1030-3) contains supplementary material, which is available to authorized users.

## Background

Hypertension is one of the most common diseases afflicting humans worldwide. It is a major risk factor for stroke, myocardial infarction, vascular disease and chronic kidney disease. Hypertension may contribute to heart failure in as many as 50 to 60 % of patients. It contributes to 50 % of ischemic strokes and increases the risk of hemorrhagic stroke. Hypertension is the primary cause for myocardial fibrosis, and contributes to the pathophysiology of cardiac damage [1, 2]. Hypertension causes a sustained increase in cardiac pressure overload, triggering excessive proliferation and the accumulation of interstitial and perivascular collagen fibers that leads to myocardial fibrosis [3]. Myocardial fibrosis is an integral component of chronic heart disease [4], and it is increasingly thought to have a key role in disease progression as well [5]. Unfortunately, no specific therapies capable of halting or reversing myocardial fibrosis are currently available. Therefore, myocardial fibrosis prevention and treatment is a subject of great clinical value and public health interest.

Transforming growth factor β_1_ (TGF-β_1_) is a key regulatory factor involved in the proliferation and differentiation of fibroblasts [6]. Recent pharmacological studies targeting TGF-β_1_ have reported progress in the development of myocardial fibrosis treatments [7], but the clinical efficacy of these treatments has yet to be established.

Traditional Chinese medicine (TCM) employs mixtures of multiple herbs to treat patients. These mixtures are referred to as formulas or “Fufang”. It is currently thought that the therapeutic effects that have been demonstrated for many specific TCM therapies are mediated by multiple ingredients. Pharmacologically active ingredients have been demonstrated to be present in several of these formulas, and some of these formulas have been reported to be therapeutically effective against multiple diseases, including tumors, inflammatory conditions and cardiac diseases [8].

Spontaneously hypertensive rat (SHR) is a well-established model for studying the mechanisms of hypertrophy and heart failure associated with genetic hypertension. SHR is often used as a model of human essential hypertension-induced myocardial fibrosis [9]. The SHR develops hypertension at 2 to 6 weeks, followed by progressive myocardial fibrosis, cardiac hypertrophy, and eventual heart failure [10, 11]. Typical symptoms of myocardial fibrosis include left ventricular hypertrophy (LVH) and age-related collagen increase [12]. Several groups reported myocardial collagen concentration and elevated collagen synthesis in SHR when compared with WKY rats of similar age [13, 14]. The myocardial fibrosis and hypertrophy in SHR can be prevented using specific antihypertensive agents [15].

Numerous studies published in the Chinese medical literature have reported the successful treatment of symptoms related to high blood pressure, such as headaches and dizziness, using the herbal formulas Xue-Fu-Zhu-Yu decoction (XFZYD), Tian-Ma-Gou-Teng-Yin (TMGTY) and Wen-Dan decoction (WDD) [16, 17]. Previous studies showed that the three Chinese formulas have significant effects on a variety of hypertension caused cardiovascular diseases. For example, TMGTY can counteract myocardial fibrosis in SHR by reducing LVI and collagen content [18]. Otherwise, the effect of TMGTY on arterial pressure was through an action of TMGTY on sympathetic vasomotor activity [19]. XFZYD improves endothelial function and the state before hypertensive thrombosis in the vessels of the SHRs with hypertension and reduces the formation of myocardial collagenIand III [20]. XFZYD could also improve the ischemic necrosis and promoting the angiogenesis [21]. WDD effectively regulates the lipid metabolism, prevent and treat the hyperlipidemia induced diseases in SHR [22]. Therefore, we assume that three Chinese formulas have effect on reversion of hypertensive myocardial fibrosis in SHR. However, the underlying mechanisms of action and therapeutic effects of treatment with these herbal formulas remain unclear.

The present study aimed to investigate whether XFZYD, TMGTY or WDD were capable of reversing myocardial fibrosis in SHR and to elucidate the mechanisms underlying any therapeutic effects found.

## Methods

### Drug preparation

Traditional Chinese herb granules manufactured by Guangdong EFONG Pharmaceutical Co. Ltd. (Guangzhou, China) were purchased from the pharmacy of Southern Hospital. Captopril tablets (12.5 mg) were purchased from Bristol-Myers Squibb Co. Ltd. (Shanghai, China; Lot number: 1209031). The components comprising the TMGTY, WDD and XFZYD formulas are listed in Table 1, and are based on established quality control procedures [8, 19, 23]. After preparation, the herbal mixtures were freeze-dried. Prior to use, each 2 g of dry herbal mixture was resuspended in 1 ml distilled water.

**Table 1:** Composition of Chinese herbal formulas

Components	Source	Amount used (g)	Lot number
Tian-Ma-Gou-Teng-Yin (TMGTY)			
*Gastrodia elata Blume*	Root	9	407233 T
*Uncaria rhynchophylla (Miq.) Miq. ExHavil.*	Hook	12	404145 T
*Reynoutria multiflora (Thunb.) Moldenke*	Aerial part	9	404203 T
*Haliotis diversicolor Reeve*	Conch	18	311190 T
*Eucommia ulmoides Oliv.*	Cortex	9	407270 T
*Scutellaria baicalensis Georgi*	Root	9	409032 T
*Gardenia jasminoides J.Ellis*	Fruit	9	407018 T
*Leonurus japonicas Houtt.*	Aerial part	9	409112 T
*Viscum coloratum (Kom.) Nakai*	Stem	9	408443 T
*Achyranthes bidentata Blume*	Root	12	409081 T
*Poria cocos (Schw.) Wolf*	Sclerotium	9	406029 T
Wen-Dan decoction (WDD)			
*Pinellia ternata (Thunb.) Makino*	Tuber	9	408101 T
*Citrus × aurantium L.*	Young fruit	12	401084 T
*Citrus reticulata Blanco*	Mature pericarp	9	409424 T
*Pleioblastus amarus (Keng) Keng f.*	Stem	9	407209 T
*Glycyrrhiz auralensis Fisch.*	Rhizome	5	408447 T
*Poria cocos (Schw.) Wolf*	Sclerotium	5	408407 T
Xue-Fu-Zhu-Yu decoction (XFZYD)			
*Angelica sinensis (Oliv.) Diels*	Root	9	410141 T
*Bupleurum chinense DC.*	Root	3	407027 T
*Carthamus tinctorius L.*	Flower	9	408165 T
*Citrus aurantium L.*	Fruit	6	403011 T
*Cyathula officinalis Kuan*	Root	9	409101 T
*Glycyrrhiza glabra L.*	Root	3	408447 T
*Ligusticum striatum DC.*	Root	5	410141 T
*Paeonia lactiflora Pall.*	Root	6	407380 T
*Platycodon grandiflorus (Jacq.) A. DC.*	Root	5	408059 T
*Prunus persica (L.) Batsch.*	Seed	12	406423 T
*Rehmannia glutinosa (Gaertn.) DC.*	Root	9	403409 T

### Animal studies

Forty-five 12-wk-old male SHRs (280 – 320 g) and five age-matched male Wistar-Kyoto (WKY) rats (285–310 g) were purchased from VITAL RIVER Co. Ltd. (Beijing, China; certificate number, SCXK2012-001). SHRs were selected based on total hypertension and established myocardial fibrosis. The WKY rats were used as controls. All rats were maintained in plastic cages with soft bedding under a 12/12 h light–dark cycle. The rats received standard care and had free access to a standard diet and drinking water. The protocol of this study was reviewed and approved by the Animal Review Board of Southern Medical University (Guangzhou, China). The research was conducted under the Animal [Scientific Procedures] Act of 1986 and the institutional guidelines of Southern Medical University for the care and use of animals.

The SHRs were randomized into nine groups with five rats in each group. The group 1 rats were treated with distilled water and served as the SHR no-treatment control group. Groups 2 and 3 were treated with 6 g∙kg^−1^ and 12 g∙kg^−1^ TMGTY, respectively. Groups 4 and 5 were treated with 6 g∙kg^−1^ and 12 g∙kg^−1^ WDD, respectively. Groups 6 and 7 were treated with 6 g∙kg^−1^ and 12 g∙kg^−1^ XFZYD, respectively. Groups 8 and 9 were treated with 30 mg∙kg^−1^ and 60 mg∙kg^−1^ captopril, respectively, and were used as the positive treatment control group. Each SHR was given daily oral administration of the appropriate designated drug. The WKY control rats were also treated with distilled water as similar with the group 1 rats for the blank control. All the rats were fed under Specific Pathogen-Free (SPF) conditions, with fresh, sterilized food, vegetables and water that were changed each day. Bedding was replaced once every two days. Environmental noise was less than 60 dB, temperature was maintained between 23 and 26 °C and humidity was maintained between 40 and 70 %.

The total treatment course was 16 weeks. Rat body weight was monitored each wk and systolic blood pressure was measured before and after each experiment. All rats survived the study, and no significant abnormalities were observed. After the final treatment, rats were anesthetized using 350 mg/kg of 10 % chloral hydrate and then euthanized using cervical dislocation. The heart was quickly removed from each euthanized rat, washed with cold normal saline and then dried with filter paper. One-hundred mg of the left ventricle was removed and stored at −70 °C for further analysis. The remainder of the left ventricle was fixed in 10 % formalin for histology.

### Systolic blood pressure measurements

Rats were housed in a 37 °C chamber for 10 min for indirect systolic blood pressure measurements, then transferred to a standard blood pressure measurement apparatus that included a heating pad, acrylic restrainer, tail cuff and pulse sensor (NarcoBiosystems, Houston, TX, USA). The tail cuff was connected to a cylinder of compressed air via inlet and outlet valves that allowed steady inflation and deflation of the cuff. Tail cuff pressure was continuously recorded using a solid-state pressure sensor (Sensym, Honeywell Sensing & Control, Inc., Morristown, NJ, USA). Pulse and pressure sensor signals were amplified and then digitized with an analogy-digital board (DT16EZ, Data Translation, Inc., Marlboro, MA, USA) connected to a desktop computer. Labtech Notebook Pro (Laboratory Technology Corp., Wilmington, MA, USA) was used to control and display the procedure online. Inflation and deflation scores and the compression interval were recorded for each rat [24].

### Hydroxyproline content assay

The chloramine T assay was used to quantify hydroxyproline (HYP) in the tissues collected from the rats in this study [25]. The apex of the left ventricle of each rat was defatted and lysed. Next, the sample was centrifuged at 4000 rpm for 10 min. After centrifugation, the supernatant was mixed with fresh chloramine T for 10 min, followed by mixture with Ehrlich’s reagent at 75 °C for 20 min. Samples were then cooled, and the optical density of each sample was read at 560 nm with a microplate reader (iMark, Bio-Rad laboratories, USA). Prior to measuring each sample, the microplate reader was adjusted using a “blank” sample that was prepared using the same procedure used to prepare the experimental samples, but without any cardiac tissue in the reaction mixture. HYP concentration, expressed as micrograms per milligram of dry heart weight, was then calculated as previously described [26].

### Histological examination

The remaining ventricular tissue not used in the hydroxyproline content assay was immediately fixed in 10 % formalin, embedded in paraffin, sliced into 5 μm sections and then heated for 2 h in a 65 °C incubator. These sections were stained using the Masson trichrome method to examine myocardial interstitial fibrosis [27]. Each section was photographed at 400 x magnification using a light microscope (CX31, Olympus, Tokyo, Japan). All images were analyzed using Image-pro Plus 6.0 software (Media Cybernetics, Bethesda, MD, USA).

The percentage of collagen present in each microscopic field represented the myocardial interstitial collagen content. The accumulation of collagen content in the interstitial spaces of the left ventricle were assessed using polarized light microscopy and analyzed using Image-pro Plus 6.0 software.

### Immunohistochemical analysis of TGF-β1 expression

The middle ring of the left ventricle was fixed in 10 % neutral formalin for 24 h, embedded in paraffin and cut into 4 μm sections. Each section was incubated at 4 °C overnight with anti-TGF-β_1_ antibody (1:1000, Proteintech Co., Chicago, IL, USA) and at 37 °C for 30 min with Mo/Rb type I polymer (Genecompany, Guangzhou, China). The streptavidin-biotin-peroxidase complex technique (Histostain Plus Kit, cat. No. 85–8943; Zymed, Life Technologies, Grand Island, NY, USA) was used for immunohistochemical visualization of antibody-bound sections. Sections were observed at 400 x magnification, and TGF-β_1_ staining intensity was analyzed using Image-pro Plus 6.0 software. Twenty-five random fields per section were analyzed and combined to obtain a final value for each section.

### TGF-β_1_ mRNA expression measurement

TGF-β_1_ mRNA expression was determined using real-time reverse transcription polymerase chain reaction (RT-PCR). Total RNA was extracted from tissues using Trizol (Sigma, St Louis, MO, USA). RNA yields and purity were assessed by spectrophotometric analysis (BioPhotometer plus, Eppendorf, Germany). Total RNA (1 μg/μl) transcription was performed using an *in vitro* transcription Kit (PrimeScript RT reagent Kit Perfect Real Time, TaKaRa, Japan). RT-PCR reactions were performed with 20 μl reactions that consisted of 10 μl 2X SYBR Premix ExTaq, 0.4 μl PCR Forward primer (10 μM), 0.4 μl PCR reverse primer (10 μM), 2 μl cDNA, 0.4 μl ROX Reference Dye II and 6.8 μl double-distilled water. PCR was conducted with an initial denaturation step of 95 °C for 30 s, followed by 40 cycles of denaturation at 95 °C for 5 s and 60 °C annealing for 34 s. All RT-PCR reactions were performed in triplicate for each sample. The primer sequences were as follows:

TGF-β_1_

Forward: 5’ - CATTGCTGTCCCGTGCAGA - 3’

Reverse: 5’ - AGGTAACGCCAGGAATTGTTGCTA - 3’

β-actin

Forward: 5’ - TGACGTTGACATCCGTAAAGACC - 3’

Reverse: 5’ - GTGCTAGGAGCCAGGGCAGTAA - 3’

RT-PCR and data analysis were carried out using ABI 7500 Software 2.0.3 (Life Technologies). Values obtained for TGF-β_1_ were normalized against values obtained for β-actin, and the results were expressed as relative integrated intensity.

### Western blot analysis of TGF-β_1_

Heart tissue was homogenized in ice-cold loading buffer (pH 6.8). Total protein was extracted using RIPA Lysis Buffer (Fu De Biological Technology, Hangzhou, China). The protein was separated using a 12 % SDS-polyacrylamide gel in electrophoresis sample buffer and then transferred to PVDF membranes. After blocking membranes for 1.5 h with 5 % bovine serum albumin (BSA) in tris-buffered saline containing 0.1 % Tween-20 at pH 7.6 (TBST), the membranes were probed overnight at 4 °C with antibodies against TGF-β_1_ (1:1500, product number: ab92486, Epitomics, Burlingame, California, USA and Abcam, Cambridge, UK). After the overnight primary antibody incubation, the membranes were washed with TBST, once for 15 min and then an additional three times for 5 min each. After washing, the membranes were incubated with goat anti-rabbit horseradish peroxidase-conjugated secondary antibody (1:6000, product number: #7074, Cell Signaling Technology, Danvers, MA, USA) for 1 h at room temperature and then washed three times with TBST for a total of 30 min. Antibody-antigen complexes were visualized using enhanced chemiluminescence (Millipore, USA). Images were scanned and the results were quantified using Image-pro Plus 6.0 software.

### Statistical analysis

All data were presented as means ± standard deviation (SD). Multiple comparisons were performed using one-way ANOVA (*p* < 0.05) followed by Dunnett’s post-hoc *t*-test. All statistical analyses were performed using SPSS 13.0 software (SPSS Inc. Chicago, IL, USA).

## Results

### Effects of TCM on systolic blood pressure and body weight

Data obtained during this study were consistent with data reported by previous studies, and indicated that the systolic blood pressure of control SHRs was much higher than that of WKY rats (Table 2, *p* = 0.005). Comparisons among the SHRs treated with WDD, XFZYD, TMGTY and captopril revealed that captopril treatment alone significantly reduced systolic blood pressure (*p* = 0.032). None of the other treatments demonstrated any anti-hypertensive effect (*p* = 0.085, WDD; *p* = 0.075, XFZYD; *p* = 0.103, TMGTY).

**Table 2:** Effects of TCM on systolic blood pressure and body weight

		SHRs								
	WKY	Control	TMGTY		WDD		XFZYD		CAPTOPRIL	
			6 g∙kg^−1^	12 g∙kg^−1^	6 g∙kg^−1^	12 g∙kg^−1^	6 g∙kg^−1^	12 g∙kg^−1^	30 mg∙kg^−1^	60 mg∙kg^−1^
Systolic blood pressure (mmHg)										
Before	123 ± 13	177 ± 8^**^	173 ± 13	176 ± 9	175 ± 11	179 ± 7	172 ± 14	170 ± 16	177 ± 13	178 ± 11
After	128 ± 11	188 ± 6^**^	179 ± 8	178 ± 6	177 ± 7	180 ± 9	170 ± 12	168 ± 5	161 ± 10^* ***^	153 ± 9^* ***^
Body weight (g)										
Before	299 ± 7	310 ± 5	305 ± 9	303 ± 6	302 ± 7	306 ± 7	294 ± 11	298 ± 8	305 ± 7	302 ± 10
After	361 ± 12	363 ± 4	345 ± 10	351 ± 11	342 ± 13	336 ± 5	332 ± 10	330 ± 9	330 ± 8	329 ± 14

**p < 0.01 compared to the WKY rats, ***p < 0.05 compared to the SHR control

*p < 0.05 compared to the corresponding data before the experiment

### Effect of TCM on myocardial HYP content

**Fig. 1:**
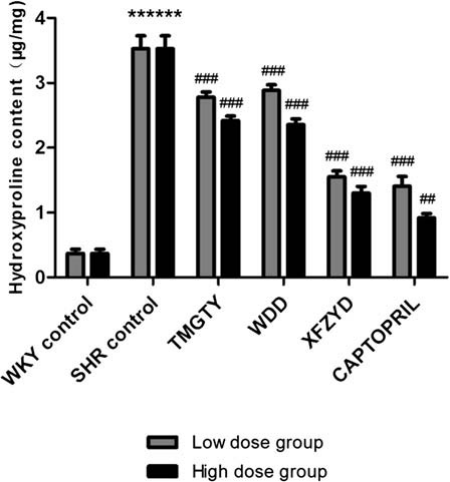
Hydroxyproline levels in the left ventricle in SHR and WKY (*n* = 3)

Myocardial HYP content levels are illustrated in Fig. 1. TMGTY, WDD, XFZYD and captopril treatment all significantly reduced the left ventricular HYP content of SHRs. The strongest effect was seen after captopril (*p* < 0.001, F = 94.431, df = 17, low-dose group; *p* = 0.003, F = 110.054, df = 17, high-dose group) and XFZYD (*p* < 0.001, low- and high-dose groups) treatment. High dose treatment groups exhibited a stronger HYP reduction than the low dose treatment groups.

### Effect of TCM on myocardial collagen

**Fig. 2:**
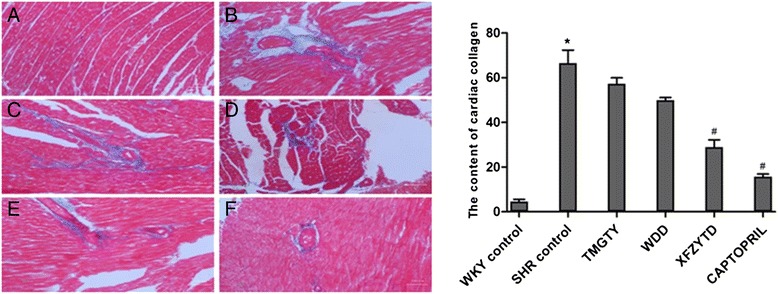
Representative micrographs of cardiac collagen in the interstitial space of the left ventricle. (*n* = 3) (**a**) WKY control, (**b**) SHR control, (**c**) SHR with 12 g∙kg^−1^ TMGTY, (**d**) SHR with 12 g∙kg^−1^ WDD, (**e**) SHR with 12 g∙kg^−1^ XFZYD and (**f**) SHR with 60 mg∙kg^−1^ captopril

Red or deep red normal muscle fibers and blue myocardial interstitial collagen fibers were observed during microscopic examination of Masson trichrome stained tissues (Fig. 2). Generally, less collagen accumulation was seen in the myocardia of WKY tissues when compared with tissues from the SHR control group (*p* = 0.041; F = 74.927, df = 17; Fig. 2). After treatment, collagen levels were significantly reduced in all treatment groups. Notably, less collagen was deposited in the SHR groups treated with the TCM formulas when compared with the collagen levels of the control SHR group, and both XFZYD (12 g∙kg^−1^, *p* = 0.018) and captopril (60 mg∙kg^−1^, *p* = 0.007) significantly reduced the post-treatment myocardial collagen levels. The observed reduction in myocardial collagen was not statistically significant after treatment with TMGTY (12 g∙kg^−1^, *p* = 0.064) or WDD (12 g∙kg^−1^, *p* = 0.092).

### Effect of TCM on myocardial TGF-β_1_

**Fig. 3:**
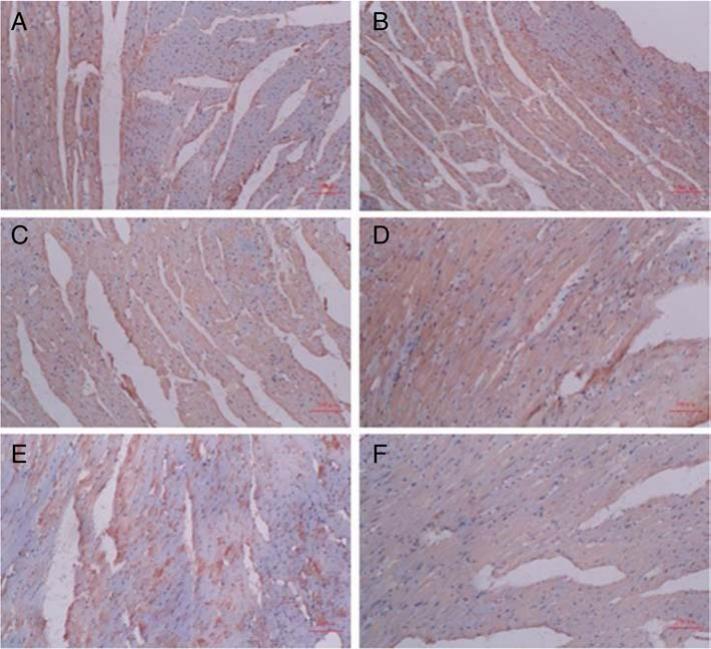
Representative micrographs of TGF-β_1_ protein expression in myocardial tissue. (*n* = 3) (**a**) WKY control, (**b**) SHR control, (**c**) SHR with 12 g∙kg^−1^ TMGTY, (**d**) SHR with 12 g∙kg^−1^ WDD, (**e**) SHR with 12 g∙kg^−1^ XFZYD and (**f**) SHR with 60 mg∙kg^−1^ captopril

TGF-β_1_ expression in the myocardial interstitium of SHR controls was significantly increased when compared with the myocardial interstitium of WKY control rats (*p* = 0.039; F = 73.108, df = 17; Fig. 3). Immunohistochemistry revealed that the captopril (60 mg∙kg^−1^, *p* = 0.021) and XFZYD (12 g∙kg^−1^, *p* = 0.034) treated groups exhibited significantly reduced brown granule deposits, suggesting that TGF-β_1_ expression was reduced in these groups when compared with the control SHR group (Table 3). The concentrations of brown granule deposits were not significantly different between the TMGTY (12 g∙kg^−1^, *p* = 0.071) and WDD (12 g∙kg^−1^, *p* = 0.078) treatment groups.

**Table 3:** Effects of TCM on myocardial TGF-β_1_

Sample	IOD	Area(μm^2^)	Density
WKY control	1839 ± 75	2290 ± 122	0.81 ± 0.07
SHR control	1225 ± 39	1018 ± 20	1.20 ± 0.01^*^
TMGTY	1306 ± 74	1179 ± 58	1.12 ± 0.12
WDD	1740 ± 46	1580 ± 85	1.11 ± 0.08
XFZYTD	921 ± 43	1009 ± 43	0.92 ± 0.09^**^
CAPTOPRIL	591 ± 60	708 ± 11	0.83 ± 0.07^**^

*p < 0.05 compared to the WKY control, **p < 0.05 compared to the SHR control

### TGF-β_1_ mRNA expression in SHR and WKY

**Fig. 4:**
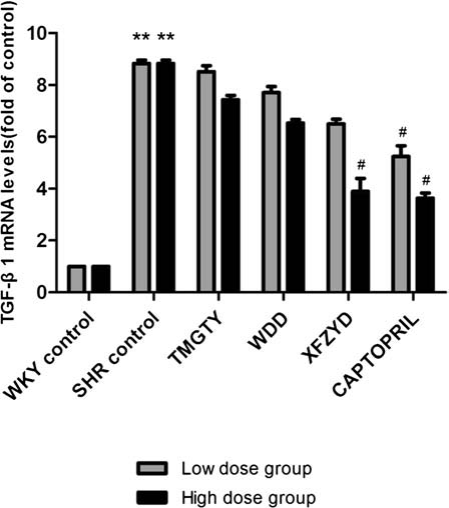
TGF-β_1_ mRNA expression in SHR and WKY. (*n* = 3)

TGF-β_1_ mRNA expression was increased in the left ventricles of control SHRs when compared with WKY control rats (*p* = 0.007; F = 152.538, df = 17, low-dose group; F = 137.812, df = 17, high-dose group; Fig. 4). Treatment with captopril (30 mg∙kg^−1^, *p* = 0.025; 60 mg∙kg^−1^, *p* = 0.009) and XFZYD (12 g∙kg^−1^, *p* = 0.031) significantly reduced TGF-β_1_ mRNA expression in a dose-dependent manner. In contrast, neither TMGTY (6 g∙kg^−1^, *p* = 0.095; 12 g∙kg^−1^, *p* = 0.076) nor WDD (6 g∙kg^−1^, *p* = 0.101; 12 g∙kg^−1^, *p* = 0.078) treatment had any effect on TGF-β_1_ mRNA expression.

### TGF-β_1_ protein expression in SHR and WKY

**Fig. 5:**
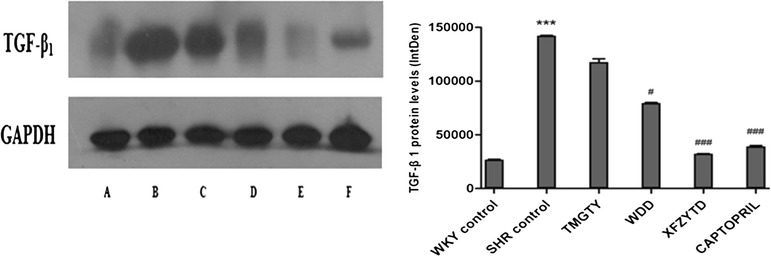
Representative TGF-β_1_ Western blots*.* (*n* = 3) (**a)** WKY control, (**b)** SHR control, (**c)** SHR with 12 g∙kg^−1^ TMGTY, (**d)** SHR with 12 g∙kg^−1^ WDD, (**e)** SHR with 12 g∙kg^−1^ XFZYD and (**f)** SHR with 60 mg∙kg^−1^ captopril

TGF-β_1_ protein expression differed significantly between the control SHR group and the WKY control group (*p* < 0.001; F = 1408.471, df = 17; Fig. 5). Furthermore, TGF-β_1_ protein expression in SHRs was significantly decreased in the captopril (60 mg∙kg^−1^) and XFZYD (12 g∙kg^−1^) treatment groups when compared with the control SHR group (*p* < 0.001). Treatment with WDD (12 g∙kg^−1^) also markedly reduced TGF-β_1_ protein expression when compared with the control SHR group (*p* = 0.036). In contrast, TMGTY (12 g∙kg^−1^) treatment was not associated with a statistically significant decrease in TGF-β_1_ protein expression (*p* = 0.064).

## Discussion

Systemic hypertension is a highly prevalent chronic disease and a significant determinant of cardiovascular morbidity and mortality [28, 29]. It is a primary cause of myocardial fibrosis, which in turn plays a key role in the pathophysiology of hypertension-induced cardiac damage [30]. Myocardial fibrosis is the excessive deposition of collagen fibers in myocardial tissue [31], and has been closely associated with hypertension, myocardial infarction, atherosclerosis and diabetes. Thus, the inhibition of early hypertension may delay ventricular remodeling and heart failure [32].

Captopril is an angiotensin-converting enzyme inhibitor that is used as an anti-hypertensive agent. It is also ameliorates myocardial fibrosis [33], and is used to treat certain types of congestive heart failure [34]. In this study, we compared the efficacy of captopril with three different Chinese herbal formulas derived from TCM for the treatment of myocardial fibrosis in SHRs.

The baseline blood pressure measured in this study was much higher in SHRs when compared with the baseline blood pressure in WKY rats. WDD, XFZYD and TMGTY treatment had no effect on the systolic blood pressure, whereas captopril treatment significantly reduced the systolic blood pressure measured in SHRs. XFZYD treatment at 12 g∙kg^−1^ each day for 16 weeks effectively inhibited myocardial fibrosis and decreased HYP in SHR myocardial tissue. Our results suggest that the XFZYD induced reduction in fibrosis and HYP content was independent of blood pressure, providing a theoretical basis for the clinical application of XFZYD.

Cardiac hypertrophy and interstitial fibrosis are common outcomes after cardiac injury and overload [35, 36]. TGF-β overexpression is a clear factor underlying tissue fibrosis development in numerous human diseases. Furthermore, TGF-β plays a crucial role in pathogenic processes associated with cardiac remodeling and fibrosis [37], and the over expression of cardiac TGF-β_1_ is associated with both fibrosis and hypertrophy [38].

Using immunohistochemical, RT-PCR and Western blot analysis, we confirmed that the expression of TGF-β_1_ mRNA and protein was reduced in SHRs after 16 weeks of daily treatment with 12 g∙kg^−1^ XFZYD. We conclude that XFZYD inhibited fibrosis by disrupting the TGF-β signaling pathway. Other TCM treatments had no significant effects.

XFZYD is a classic formula in TCM clinical for treatment of heart disease, our study found that XFZYD has the effect of reverse hypertensive myocardial fibrosis and the mechanism may be associated with TGF-β_1_ signaling pathways. So we speculate that there are some active components in the formula of XFZYD to ameliorate hypertensive myocardial fibrosis through TGF-β_1_ signaling pathways. Studies show that XFZYD not only can inhibit myocardial fibroblasts proliferation, but also to disturb the action of collagen secretion by myocardial fibroblasts [39]. Ferulic acid sodium is the main active ingredient in Angelica sinensis (Oliv.) Diels, the structure contains phenolic hydroxyl, which has the function of antioxidant and antiatherosclerosis. Ferulic acid sodium can suppress the cardiac fibroblasts proliferation induced by AngII, which exhibit a dose-dependent effect, the mechanism may be associated with lower TGF-β_1_ protein expression [40]. Ligustrazine is the main component of Ligusticum striatum DC., which can block and delay the development of myocardial fibrosis in chronic pressure overload rats and also inhibit the myocardial fibroblasts induced by AngII [41]. Carthamin yellow is extracted from Carthamus tinctorius L., which can inhibit AngII and ACE and also play a good role in cardiovascular protection [42]. Amygdalin in Prunus persica (L.) Batsch has the effect of ameliorate hepatic fibrosis [43]. Paeoniflorin in Paeonia lactiflora Pall. has protective effect on myocardium in rats [44]. The existing research results are consistent with our conclusion, which can support each other. We can infer that the effective components of XFZYD formula to ameliorate myocardial fibrosis through TGF-β_1_ signaling pathways may have close relationship with ferulic acid sodium, ligustrazine, carthamin yellow, amygdalin and paeoniflorin which are extracted respectively from Angelica sinensis (Oliv.) Diels, Ligusticum striatum DC., Carthamus tinctorius L., Prunus persica (L.) Batsch and Paeonia lactiflora Pall.. We plans to research the demolition party of XFZYD in next step, and focus on the relationship between each active ingredient and TGF-β_1_ signaling pathway, in order to screen out the exact active ingredients to reverse hypertensive myocardial fibrosis in XFZYD.

## Conclusion

Among the three TCM formulas tested in this study, we found that only XFZYD was capable of reversing myocardial fibrosis. We also found that XFZYD treatment decreased hypertension-induced cardiac fibrosis independent of systolic blood pressure. Future clinical studies are needed to evaluate the therapeutic benefit of XFZYD in patients with hypertension. This study demonstrates that XFZYD decreases cardiac fibrosis via TGF-β_1_ signaling pathway.

## Additional files

Additional file 1: **The protocol of animal experimental.** (DOCX 796 kb)

Additional file 2: **The approval of the Southern Medical University Animal Cave and Use Committee.** (DOCX 1607 kb)

Additional file 3: **The constitution of the Southern Medical University Animal Cave and Use Committee.** (DOCX 3226 kb)

Additional file 4: **The staff of the Southern Medical University Animal Cave and Use Committee.** (DOCX 658 kb)

Additional file 5: **The HPLC results for the quality control of the herbs.** (PDF 27 kb)

## Abbreviations

CVF: Collagen volume fraction
SBP: Systolic blood pressure
SHR: Spontaneously hypertensive rat
TCM: Traditional Chinese Medicine
TMGTY: Tian-Ma-Gou-Teng-Yin
WDD: Wen-Dan decoction
WKY: Wistar-Kyoto rats
XFZYD: Xue-Fu-Zhu-Yu decoction
