# Supplementary figures and images for: Ameliorative effects of Xue-Fu-Zhu-Yu decoction, Tian-Ma-Gou-Teng-Yin and Wen-Dan decoction on myocardial fibrosis in a hypertensive rat mode
Source: BMC Complement Altern Med. 2016 Feb 6;16:56. doi: 10.1186/s12906-016-1030-3 (PMC4744408; doi:10.1186/s12906-016-1030-3)

**Animal Experimental Protocol**


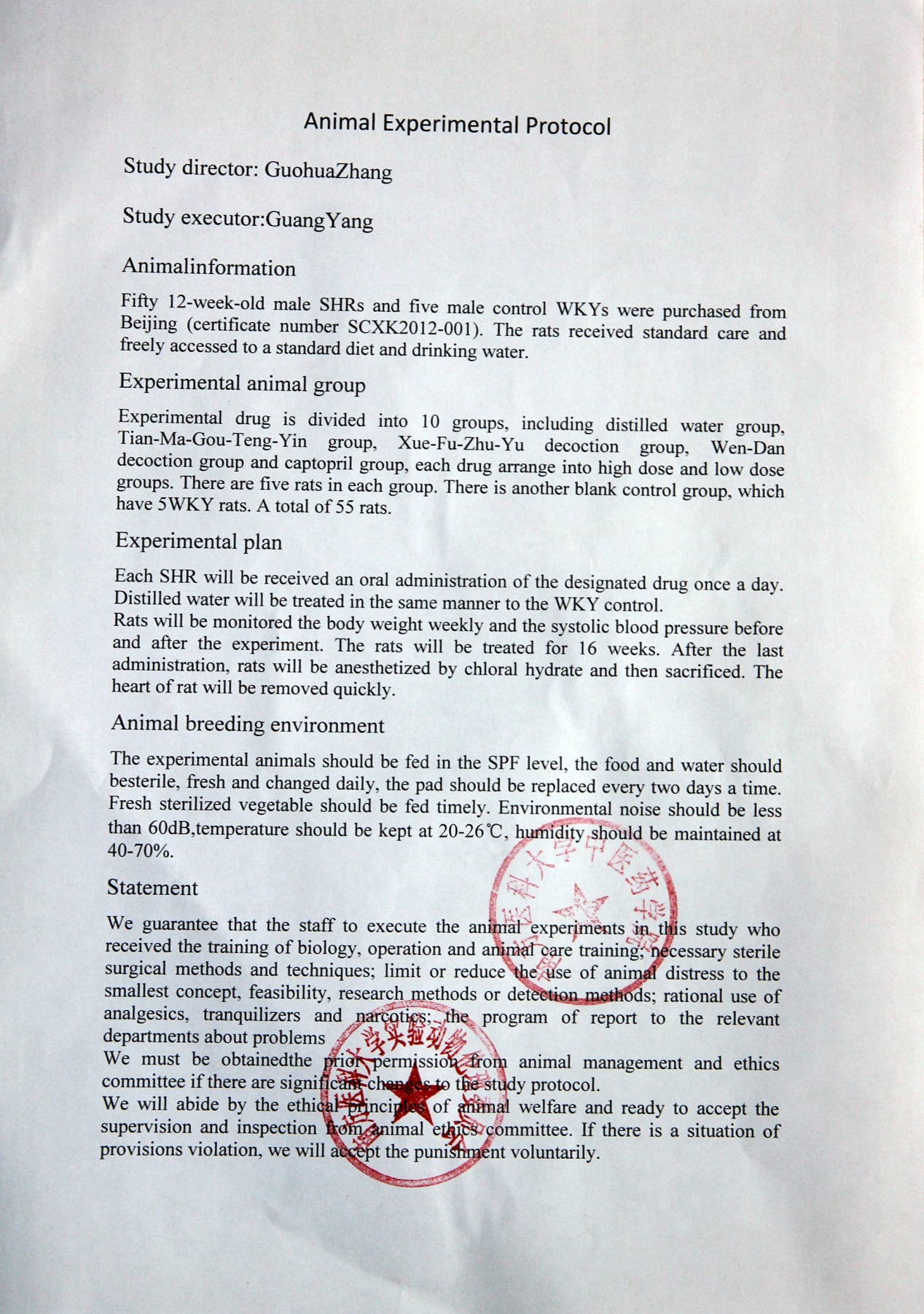

Supplement: Supplementary file 1 — The protocol of animal experimental. (DOCX 796 kb) [file 12906_2016_1030_MOESM1_ESM.docx]

**Approval of the Southern Medical University Animal Cave and Use Committee**


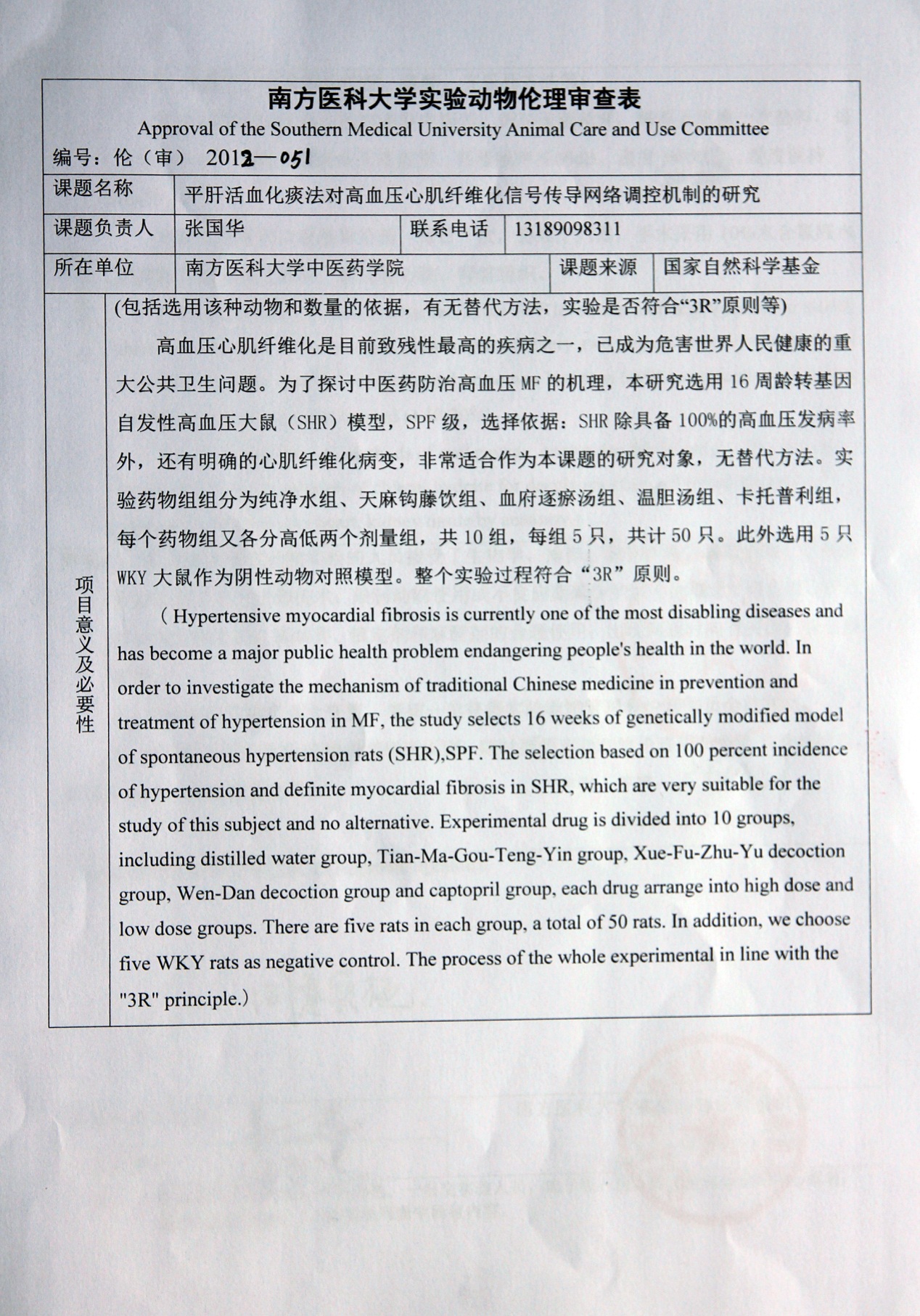


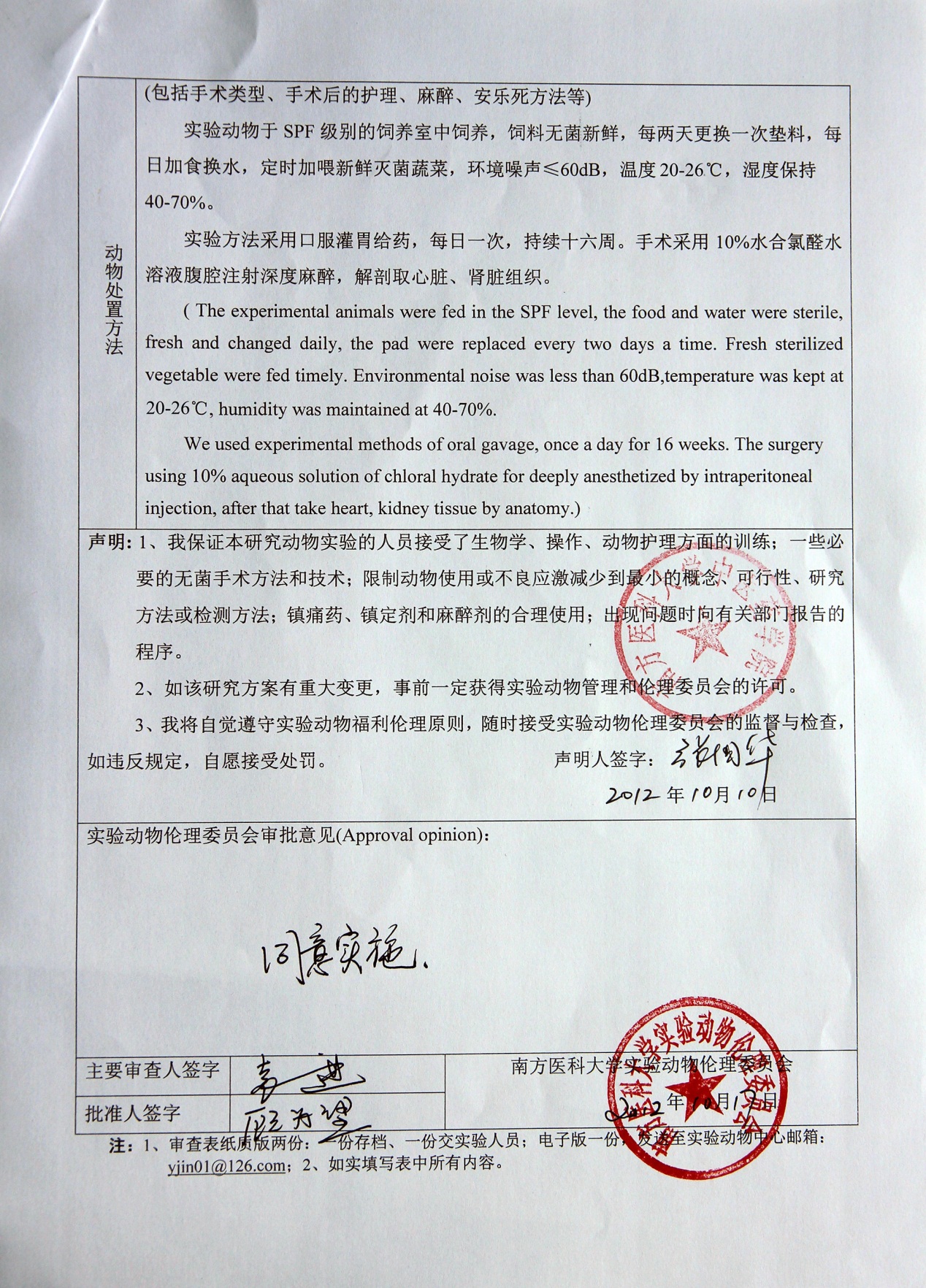

Supplement: Supplementary file 2 — The approval of the Southern Medical University Animal Cave and Use Committee. (DOCX 1607 kb) [file 12906_2016_1030_MOESM2_ESM.docx]

**Constitution of the Southern Medical University Animal Cave and Use Committee**


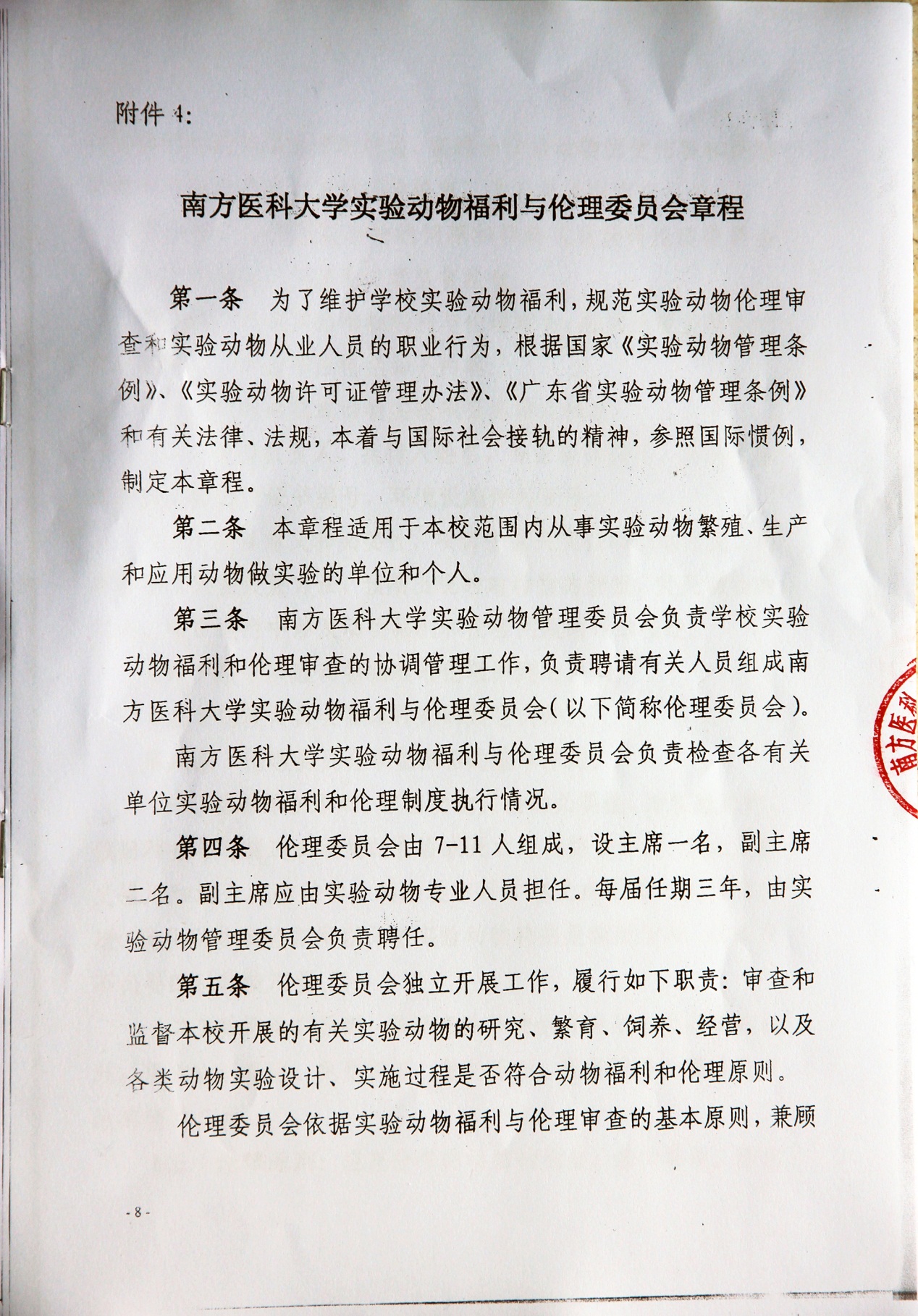


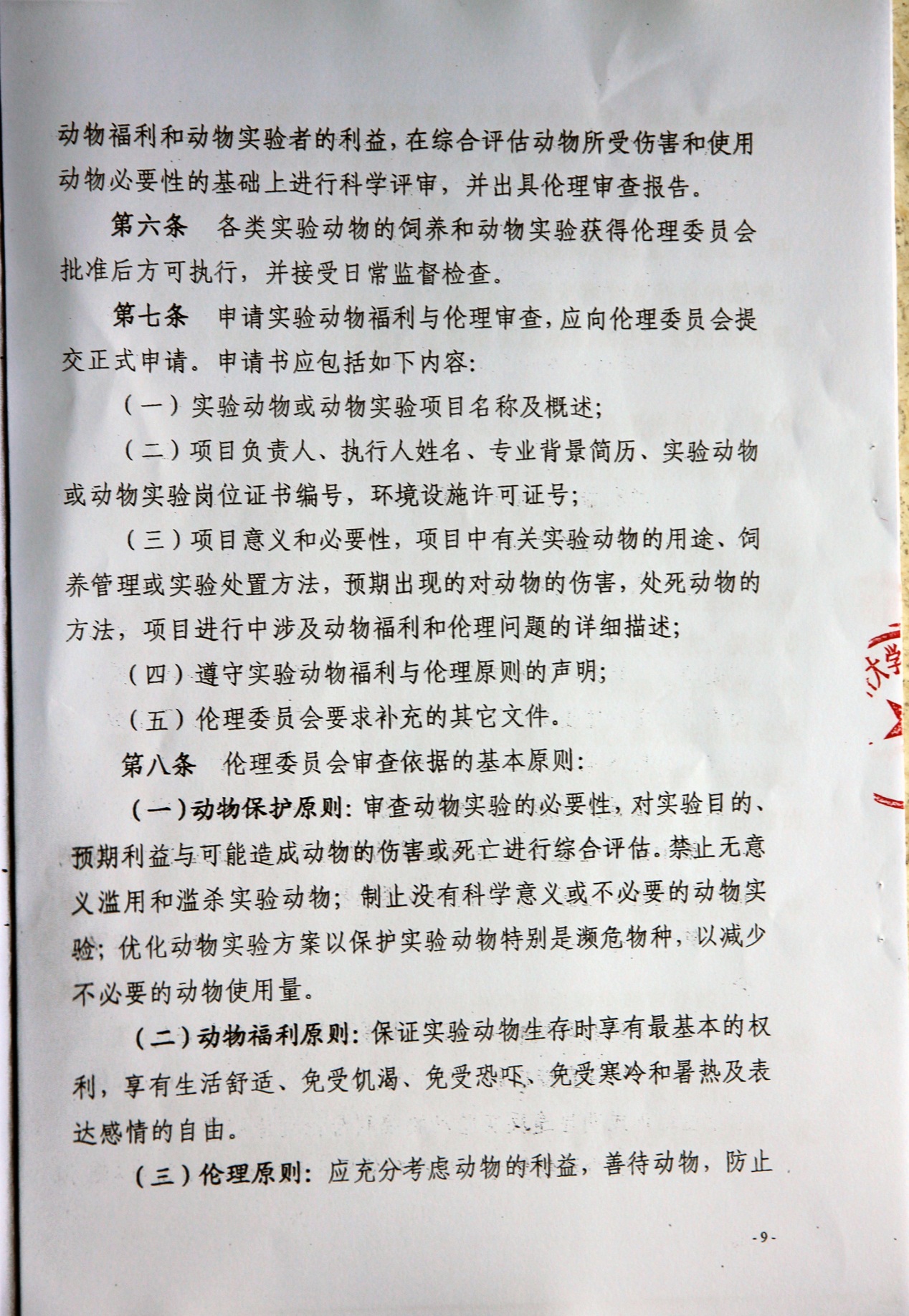


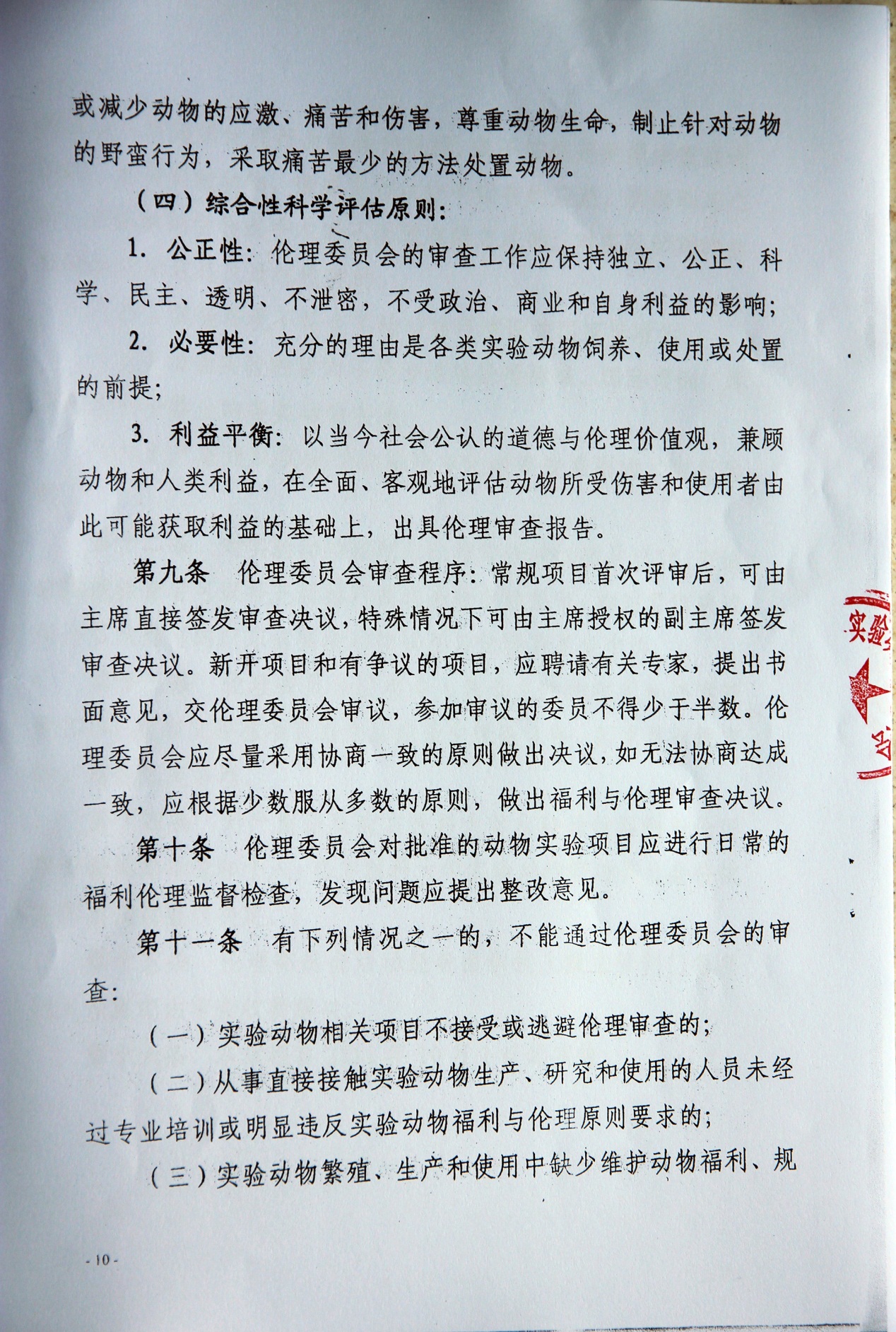


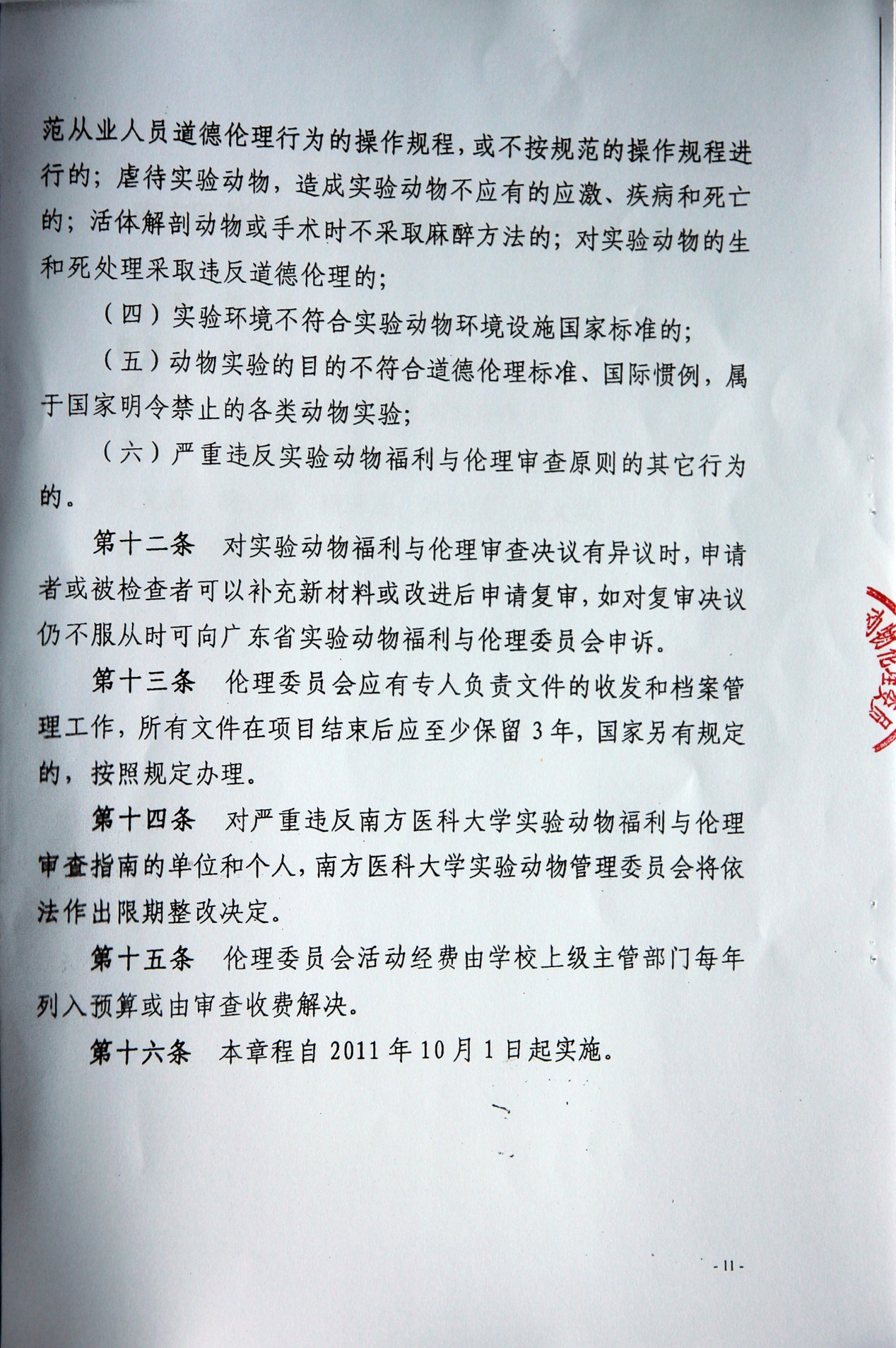

Supplement: Supplementary file 3 — The constitution of the Southern Medical University Animal Cave and Use Committee. (DOCX 3226 kb) [file 12906_2016_1030_MOESM3_ESM.docx]

**Staff of the Southern Medical University Animal Cave and Use Committee**


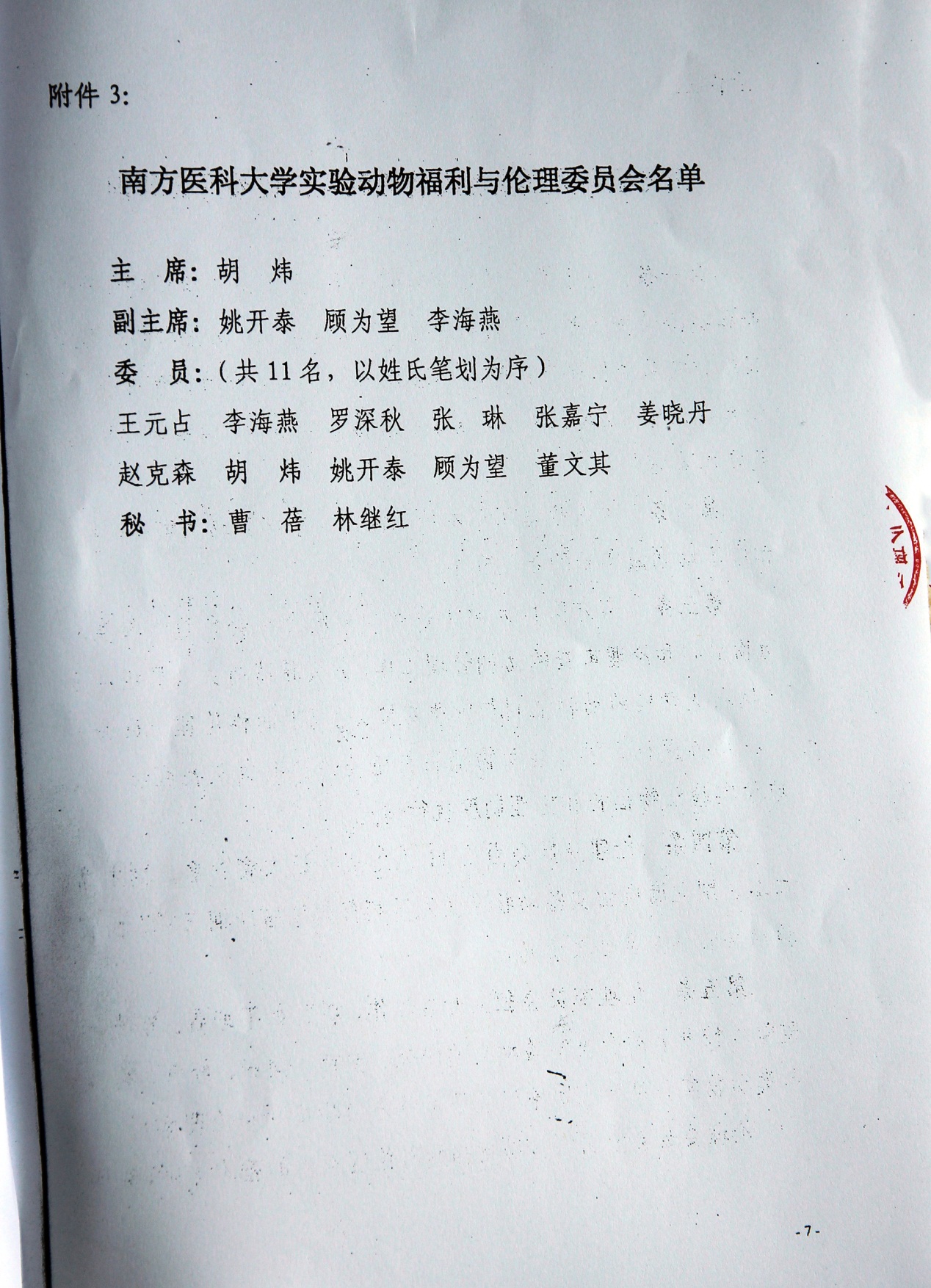

Supplement: Supplementary file 4 — The staff of the Southern Medical University Animal Cave and Use Committee. (DOCX 658 kb) [file 12906_2016_1030_MOESM4_ESM.docx]
