# Supplementary material for: Ameliorative effects of Xue-Fu-Zhu-Yu decoction, Tian-Ma-Gou-Teng-Yin and Wen-Dan decoction on myocardial fibrosis in a hypertensive rat mode
Source: BMC Complement Altern Med. 2016 Feb 6;16:56. doi: 10.1186/s12906-016-1030-3 (PMC4744408; doi:10.1186/s12906-016-1030-3)
Supplement: Supplementary file 5 — The HPLC results for the quality control of the herbs. (PDF 27 kb) [file 12906_2016_1030_MOESM5_ESM.pdf]

HPLC results for the quality control of the herbs

We found that only XFZYD was capable of reversing myocardial fibrosis among the three TCM formulas tested in this study. Further we used HPLC to analyze the quality of the six different batches of XFZYD used in the experiment. As it can be seen from the figure 1, the peak trends of main components are basically consistent from six batches herbs. Then we use the traditional Chinese medicine fingerprint similarity evaluation system (version: 2004A), all the similitude index are more than 0.9 (Table 1). Therefore, we conclude that the characteristics of the 6 batches of herbs used in the experiment are basically no difference, the quality of the herb is stable and controllable. The results from this experiment is reliable and reproducible.

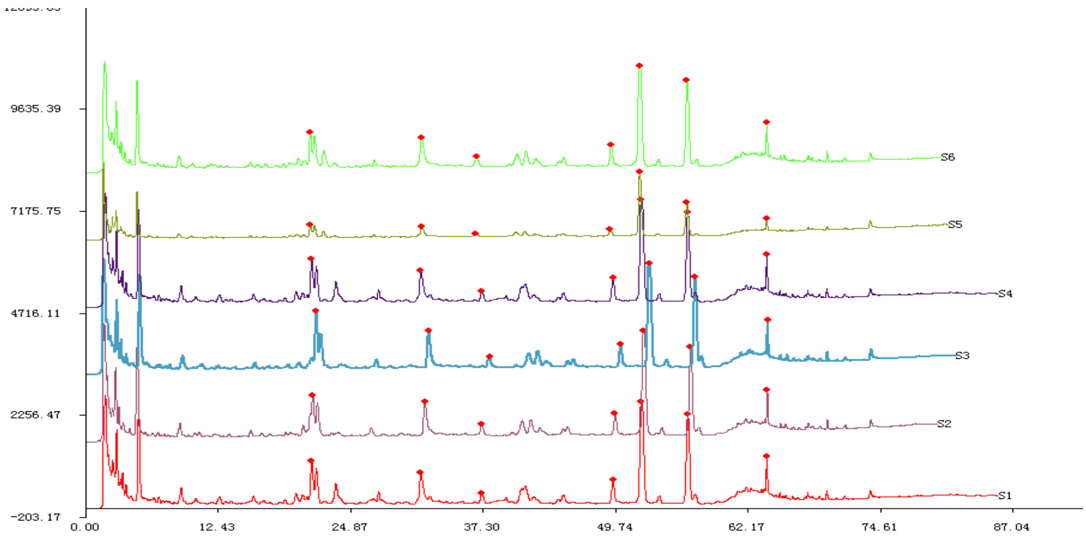

Figure 1. Comparison chart of traditional Chinese medicine fingerprint from six batches of XFZYD

Table 1. The similitude index of six batches of XFZYD

Batch number	1	2	3	4	5	6
Similitude index	0.95	0.967	0.967	0.95	0.972	0.973
